# Associations Among Emotional Intelligence, Job Stress, and Clinical Decision‐Making in Prehospital Emergency Staff: A Cross‐Sectional Study

**DOI:** 10.1002/hsr2.72885

**Published:** 2026-07-25

**Authors:** Payam Emami, Mahsa Sadafi, Ameneh Marzban, Nioosha Fatahi

**Affiliations:** ^1^ Department of Emergency Medical Sciences, School of Paramedical Sciences Kurdistan University of Medical Sciences Sanandaj Iran; ^2^ Education and Research Unit, Emergency Medical Service and Disaster Management Center, Vice Chancellor for Clinical Affairs Kurdistan University of Medical Sciences Sanandaj Iran; ^3^ Department of Health in Disasters and Emergencies, School of Health Management and Information Sciences Iran University of Medical Sciences Tehran Iran

**Keywords:** clinical decision‐making, emotional intelligence, job stress, prehospital emergency staff

## Abstract

**Background and Aims:**

Prehospital emergency personnel work in highly stressful and unpredictable environments where job stress may affect performance and clinical decision‐making. Emotional intelligence has been proposed as a personal resource that may help individuals manage stress and improve decision‐making; however, evidence in prehospital emergency settings remains limited and inconsistent. Therefore, this study aimed to examine the relationships between emotional intelligence, job stress, and clinical decision‐making among prehospital emergency staff in Kurdistan, Iran.

**Methods:**

This descriptive–correlational study was conducted among 250 prehospital emergency medical staff in Kurdistan Province, Iran, from August to December 2024. Data were collected using standardized instruments, including a demographic questionnaire, the Schutte Self‐Report Emotional Intelligence Test (SSEIT), the Health and Safety Executive (HSE) Job Stress Questionnaire, and a Clinical Decision‐Making questionnaire. Statistical analyses were performed using SPSS version 27. Descriptive statistics were used to summarize the data, while Pearson correlation and multiple linear regression analyses were conducted to examine relationships among variables. A *p*‐value of < 0.05 was considered statistically significant.

**Results:**

Participants reported moderate levels of job stress (122.10 ± 14.78), emotional intelligence (110.88 ± 14.52), and clinical decision‐making (80.62 ± 11.35). Inferential analyses showed no statistically significant associations between emotional intelligence or job stress and clinical decision‐making (*p* > 0.05). However, a weak positive correlation was observed between emotional intelligence and job stress (*r* = 0.160, *p* = 0.031). In regression analyses, neither emotional intelligence nor job stress significantly predicted clinical decision‐making, explaining a very small proportion of variance (R^2^ = 0.019).

**Conclusion:**

No robust associations were found between emotional intelligence or job stress and clinical decision‐making among prehospital emergency staff. Although a weak positive correlation was observed between emotional intelligence and job stress, this finding was not reflected in predictive models. Given the cross‐sectional design and the limited explanatory power of the regression model, causal inferences cannot be made. Future longitudinal studies are warranted to explore additional individual, organizational, and contextual factors that may better explain variations in clinical decision‐making in prehospital emergency settings.

## 1 Background

Job‐related stress is known to decrease productivity and hinder adaptability, while also contributing to higher levels of employee dissatisfaction. Job stress occurs when occupational demands exceed an individual's coping capacity, resulting in adverse psychological and behavioral outcomes [1]. Prolonged exposure to such stressors may negatively affect both professional performance and personal life functioning. In healthcare settings, particularly in emergency medical services (EMS), psychological stress is intensified due to the unpredictable nature of the work environment, high patient acuity, and resource limitations. Prehospital emergency medical staff frequently encounter traumatic events, mass casualty incidents, and critically ill patients, requiring rapid response and sustained performance under pressure. These conditions make EMS one of the most stressful occupational environments in the healthcare system [2, 3]. In low‐ and middle‐income settings such as Iran, contextual factors including limited resources, high workload, and sociocultural expectations may further exacerbate occupational stress, highlighting the importance of context‐specific investigations [1, 4, 5]

To manage occupational stress effectively, psychological and emotional resources are essential. Emotional intelligence (EI) is considered one of the key personal resources that may enhance coping capacity and adaptive responses to workplace stressors. EI refers to the perceived ability to recognize, regulate, and use emotions effectively [6]. In this study, emotional intelligence was measured using the Schutte Self‐Report Emotional Intelligence Test (SSEIT), which is aligned with a trait‐based model of EI. Therefore, EI was conceptualized as a trait construct reflecting self‐perceived emotional competencies rather than objectively measured cognitive abilities. The Job Demands–Resources (JDR) model provides a useful theoretical framework for understanding the role of EI in occupational settings. According to this model, job demands such as emotional workload and high‐stress environments contribute to strain, whereas personal resources such as emotional intelligence can buffer the negative effects of stress and promote adaptive functioning. In EMS contexts, emotional demands are particularly high, as staff must manage emotionally charged situations, critical illness, and patient suffering, making EI a relevant protective factor [7, 8]. Another critical outcome in EMS practice is clinical decision‐making, which refers to the process of selecting the most appropriate action among available alternatives based on patient assessment, clinical reasoning, and situational analysis. Effective clinical decision‐making is essential for ensuring patient safety, improving care quality, and reducing medical errors [9]. In prehospital emergency care, personnel must make rapid and accurate decisions in unstable and life‐threatening conditions. Poor decision‐making may lead to delayed treatment, deterioration of patient condition, or even mortality [10].

Recent evidence suggests that emotional intelligence may positively influence clinical decision‐making by improving emotional regulation, stress tolerance, and cognitive clarity under pressure. Individuals with higher EI are better able to manage emotional responses, maintain focus in critical situations, and communicate effectively with patients and colleagues, thereby enhancing clinical performance and care quality [11, 12]. Additionally, job stress has been shown to impair cognitive functioning and decision quality, further emphasizing the interaction between psychological resources and occupational demands in EMS settings [13].

Although previous studies have examined emotional intelligence in relation to job stress, burnout, and job performance among healthcare workers, limited research has focused specifically on prehospital emergency medical staff [7, 14]. Evidence regarding the associations among emotional intelligence, job stress, and clinical decision‐making in Iranian EMS personnel remains limited. Therefore, this study aimed to investigate these associations among prehospital emergency personnel in Kurdistan, Iran, to provide context‐specific evidence on factors influencing clinical decision‐making in EMS settings.

## 2 Methods

### 2.1 Study Design and Setting

This descriptive cross‐sectional study was conducted among prehospital emergency medical service (EMS) staff in Kurdistan Province, Iran, between August and December 2024. The study was performed in EMS bases across the province, including both urban and interurban (roadside) stations.

### 2.2 Participants, Sampling and Sample Size

Participants were selected using a stratified quota sampling method with random selection within each stratum. First, all EMS bases in Kurdistan Province were identified and categorized into two strata: urban and interurban stations. The sampling frame consisted of all eligible EMS personnel working in these stations, based on the provincial EMS administrative registry. The sample allocated to each stratum was determined proportionally according to the number of personnel employed in each category.

Within each stratum, eligible participants were selected through simple random sampling using a computer‐generated random number list. Recruitment continued until the required sample size for each stratum was achieved. The sample size was determined based on a previous similar study [15] and using standard sample size estimation methods at a 95% confidence level (*Z* = 1.96). The initial sample size was then adjusted by 10% to account for potential non‐response and incomplete questionnaires, resulting in a final target sample size of 250 participants. Inclusion criteria included at least 2 years of work experience in prehospital emergency care, absence of self‐reported psychological disorders, and willingness to participate in the study. Psychological disorders were screened through self‐report during enrollment. Individuals reporting a history of diagnosed psychiatric disorders or current psychiatric medication use were excluded. A total of 280 EMS staff were approached, of whom 265 were assessed for eligibility. Fifteen individuals were excluded due to failure to meet inclusion criteria or refusal to participate. Of the eligible participants, 255 agreed to participate and completed the questionnaires. Five questionnaires were excluded because of incomplete data, resulting in a final sample of 250 participants. Therefore, the final response rate among eligible participants was 98.0%.

### 2.3 Variables and Instruments

The primary outcome variable was clinical decision‐making ability, measured using the Clinical Decision‐Making Questionnaire. Emotional intelligence and job stress were considered the main independent predictor variables. Demographic characteristics, including age, gender, education level, marital status, employment type, work station, overtime hours, and years of experience, were treated as potential confounding variables. Data were collected using four instruments: a Demographic Characteristics, the Clinical Decision‐Making Questionnaire, the Health and Safety Executive (HSE) Job Stress Questionnaire, and the Emotional Intelligence Questionnaire.

Emotional intelligence was assessed using the Schutte Self‐Report Emotional Intelligence Test (SSEIT), originally developed by Schutte et al [16]. This instrument consists of 33 items rated on a five‐point Likert scale ranging from 1 (strongly disagree) to 5 (strongly agree), with total scores ranging from 33 to 165. Higher scores indicate higher perceived emotional intelligence. The scale evaluates dimensions such as emotional perception, emotional regulation, and utilization of emotions. In the present study, emotional intelligence was conceptualized as a trait‐based construct, reflecting self‐perceived emotional abilities rather than performance‐based emotional skills.

The Health and Safety Executive (HSE) Job Stress Questionnaire includes 35 items evaluating occupational stress across several domains, including demand, control, managerial support, peer support, relationships, role clarity, and organizational change. Total scores range from 35 to 175, with higher scores reflecting greater perceived job stress. Item scores were summed to calculate the total job stress score.

The Clinical Decision‐Making Questionnaire contains 24 Likert‐scale items with total scores ranging from 24 to 120. Higher scores indicate stronger clinical decision‐making ability. Item scores were summed to generate the total clinical decision‐making score.

All questionnaires were administered using validated Persian versions. Translation and cultural adaptation of the instruments had been performed previously using forward–backward translation and expert panel review. Previous studies confirmed acceptable validity and reliability in Iranian populations. In the present study, internal consistency reliability was assessed using Cronbach's alpha coefficients. The obtained values demonstrated satisfactory reliability for Emotional Intelligence (*α* = 0.86), Job Stress (*α* = 0.79), and Clinical Decision‐Making (*α* = 0.93).

### 2.4 Data Collection Procedure

After obtaining the necessary institutional permissions, eligible participants were informed about the objectives and procedures of the study. Written informed consent was obtained before data collection. Participants completed the questionnaires anonymously during their work shifts, and completed questionnaires were collected by the researchers. To minimize selection bias, proportional stratified sampling with random selection was used. In addition, standardized instructions and anonymous questionnaire completion were employed to reduce information bias.

### 2.5 Statistical Analysis

Data were analyzed using IBM SPSS Statistics version 27. Quantitative variables were summarized using means and standard deviations, while categorical variables were described using frequencies and percentages. Pearson correlation coefficients were calculated to assess the relationships between emotional intelligence, job stress, and clinical decision‐making. Multiple linear regression analyses were conducted to examine the predictive roles of emotional intelligence and job stress on clinical decision‐making. Clinical decision‐making was entered as the dependent variable, whereas emotional intelligence and job stress were entered as independent variables. Two regression models were constructed. In the unadjusted model, emotional intelligence and job stress were simultaneously entered as predictor variables for clinical decision‐making. In the adjusted model, demographic covariates including age, gender, education level, marital status, employment type, work station, overtime hours, and years of experience were additionally entered to control for potential confounding effects. Regression coefficients (B), standardized beta coefficients (β), 95% confidence intervals, and P‐values were reported. The analyses accounted for the stratified sampling approach by proportionally including participants from both urban and interurban EMS bases. Missing data were minimal (< 5%) and were managed using listwise deletion. Prior to inferential analyses, assumptions for both unadjusted and adjusted regression models were evaluated. Normality of residuals was assessed using histograms and normal probability (P–P) plots, while linearity and homoscedasticity were examined through scatterplots of standardized residuals versus standardized predicted values. Independence of errors was confirmed using the Durbin–Watson statistic (DW = 2.04 for the unadjusted model and DW = 2.17 for the adjusted model). Multicollinearity diagnostics indicated acceptable levels of tolerance and variance inflation factor (VIF) in both models (unadjusted: Tolerance = 0.975, VIF = 1.026; adjusted: Tolerance = 0.244–0.907, VIF = 1.085–4.104). No significant violations of regression assumptions were observed.

### 2.6 Ethical Considerations

This study was approved by the Ethics Committee of Kurdistan University of Medical Sciences (Ethics Approval Number: IR. MUK. REC.1403.117). Ethical approval was obtained prior to the initiation of the study. Participation was entirely voluntary, and written informed consent was obtained from all participants before enrollment. Participants were assured of the confidentiality and anonymity of their responses and were informed that they could withdraw from the study at any time without any penalty or consequences.

## 3 Results

A total of 250 participants were included in the study. The mean job stress score was 122.10 ± 14.78. The mean emotional intelligence score was 110.88 ± 14.52, and the mean clinical decision‐making score was 80.62 ± 11.35. Most participants were male (91.6%), aged 22–31 years (49.6%), and married (61.6%). Nearly half (46.8%) worked rotational shifts, and 54.4% were employed in urban stations. The majority held a bachelor's degree (64.4%) and were permanent employees (48.8%). In addition, 67.6% reported 30–80 h of overtime per month, and 33.2% had 5–10 years of work experience.

Independent t‐test and one‐way ANOVA were used as appropriate to examine differences in emotional intelligence, job stress, and clinical decision‐making across demographic and occupational variables (Table 1). No statistically significant differences were observed between groups (all *p* > 0.05). Based on descriptive statistics and the predefined scoring ranges of the instruments, participants demonstrated moderate levels of job stress, moderately high emotional intelligence, and moderate clinical decision‐making ability (Table 2).

**Table 1:** Frequency distribution, mean, and standard deviation of job stress, emotional intelligence, and clinical decision‐making scores based on demographic characteristics.

Variable	Category	*n* (%)	Job stress (Mean ± SD)	*p*	Emotional intelligence (Mean ± SD)	*p*	Clinical decision‐making (Mean ± SD)	*p*
Gender	Male	229 (91.6)	122.00 ± 14.87	0.68	113.00 ± 14.52	0.88	80.00 ± 11.35	0.95
	Female	21 (8.4)	122.14 ± 14.92		110.86 ± 14.57		80.62 ± 11.39	
Age	22–31	124 (49.6)	122.77 ± 13.56	0.42	111.00 ± 14.04	0.47	80.39 ± 8.94	0.81
	32–41	100 (40.0)	120.34 ± 14.86		109.69 ± 14.94		80.41 ± 14.44	
	> 42	26 (10.4)	125.71 ± 20.50		115.00 ± 15.43		82.50 ± 8.07	
Shift type	Morning/Evening	108 (43.2)	122.20 ± 11.79	0.91	108.66 ± 13.84	0.59	80.13 ± 11.59	0.18
	Night	25 (10.0)	124.80 ± 22.12		106.00 ± 18.04		71.60 ± 5.12	
	Rotational	117 (46.8)	121.97 ± 15.01		111.37 ± 14.52		81.06 ± 11.41	
Education	Associate	79 (31.6)	121.85 ± 14.16	0.47	109.53 ± 13.93	0.71	79.71 ± 10.11	0.11
	Bachelor	161 (64.4)	121.86 ± 14.18		111.56 ± 15.14		81.82 ± 11.47	
	Master	10 (4.0)	128.85 ± 25.93		112.42 ± 12.14		73.00 ± 15.92	
Employment	Permanent	122 (48.8)	122.92 ± 15.33	0.74	114.26 ± 12.46	0.18	79.81 ± 10.77	0.84
	Contract	75 (30.0)	122.34 ± 15.07		110.59 ± 14.32		81.34 ± 12.88	
	Corporate	53 (21.2)	118.50 ± 9.15		105.16 ± 17.48		79.11 ± 12.96	
Marital status	Single	96 (38.4)	121.31 ± 13.10	0.63	109.29 ± 13.71	0.32	81.70 ± 9.20	0.71
	Married	154 (61.6)	122.56 ± 15.89		111.82 ± 14.97		80.34 ± 12.49	
Work station	Urban	136 (54.4)	121.56 ± 15.03	0.64	111.10 ± 16.45	0.84	79.97 ± 12.52	0.46
	Roadside	114 (45.6)	122.75 ± 14.78		110.61 ± 11.90		81.40 ± 9.79	
Overtime (h)	< 30	23 (9.2)	116.92 ± 16.96	0.41	115.84 ± 15.71	0.39	79.00 ± 12.17	0.40
	30–80	169 (67.6)	122.52 ± 15.12		110.01 ± 14.19		80.00 ± 11.95	
	> 80	58 (23.2)	123.00 ± 13.17		111.41 ± 15.03		82.96 ± 8.88	
Experience	< 5	74 (29.6)	118.58 ± 7.86	0.06	109.19 ± 14.05	0.48	80.19 ± 8.44	0.39
	5–10	83 (33.2)	124.60 ± 17.48		109.60 ± 13.10		78.78 ± 13.75	
	11–15	47 (18.8)	118.80 ± 10.96		113.50 ± 13.92		83.23 ± 11.56	
	> 15	46 (18.4)	126.87 ± 20.04		113.37 ± 18.27		82.04 ± 10.17	

**Table 2:** Mean and standard deviation of the variables under study.

Variable	Frequency (n)	Score range	Mean ± SD
Job stress	250	35–175	122.10 ± 14.78
Emotional intelligence	250	33–165	110.88 ± 14.52
Clinical decision‐making	250	24–120	80.62 ± 11.35

Pearson correlation analysis showed no statistically significant relationships between emotional intelligence and clinical decision‐making (*r* = 0.121, *p* = 0.079), or between job stress and clinical decision‐making (*r* = −0.048, *p* = 0.290). A weak but statistically significant positive correlation was observed between emotional intelligence and job stress (*r* = 0.160, *p* = 0.031) (Table 3).

**Table 3:** Descriptive statistics and correlation matrix of study variables.

Relationship	*r*	*p*
Job stress – Emotional intelligence	0.16	0.031
Job stress – Clinical decision‐making	−0.048	0.29
Emotional intelligence – Clinical decision‐making	0.121	0.079

In the unadjusted multiple linear regression model, the overall model was not statistically significant, F (2, 247) = 2.23, *p* = 0.109, explaining 1.8% of the variance in clinical decision‐making (R^2^ = 0.018, Adjusted R^2^ = 0.010). Emotional intelligence was not a significant predictor of clinical decision‐making (B = −0.036, 95% CI: −0.137 to 0.064, *p* = 0.477). Job stress showed a small positive association with clinical decision‐making; however, this relationship did not reach statistical significance (B = 0.111, 95% CI: −0.006 to 0.217, *p* = 0.039), and should be interpreted with caution given the overall non‐significant model and sensitivity across analytical specifications. In the adjusted model, which included sociodemographic and occupational covariates (age, sex, marital status, education level, employment status, shift work, and work experience), the overall regression model remained not statistically significant, F (11, 238) = 2.61, *p* = 0.104, explaining 10.8% of the variance in clinical decision‐making (R^2^ = 0.108, Adjusted R^2^ = 0.066). Emotional intelligence (B = −0.083, 95% CI: −0.184 to 0.019, *p* = 0.109) and job stress (B = 0.093, 95% CI: −0.012 to 0.198, *p* = 0.083) were not statistically significant predictors in the adjusted model, although both variables showed non‐significant trends toward association with clinical decision‐making (Table 4).

**Table 4:** Multiple linear regression analysis predicting clinical decision‐making.

Predictor	Model 1 (Unadjusted) B (95% CI)	*p*	Model 2(Adjusted) B (95% CI)	*p*
Job stress	0.111 (−0.006, 0.217)	0.039	0.093 (−0.012, 0.198)	0.083
Emotional intelligence	−0.036 (−0.137, 0.064)	0.477	−0.083 (−0.184, 0.019)	0.109

*Note*: Model 1 (Unadjusted): F(2, 247) = 2.23, *p* = 0.109, R^2^ = 0.018, Adjusted R^2^ = 0.010. Model 2 (Adjusted): F(11, 238) = 2.61, *p* = 0.104, R^2^ = 0.108, Adjusted R^2^ = 0.066. Values are presented as unstandardized regression coefficients (B) with 95% confidence intervals. Model 2 is adjusted for age, sex, marital status, education level, employment status, shift work, and work experience.

## 4 Discussion

This study examined the relationships between job stress, emotional intelligence, and clinical decision‐making among prehospital emergency medical staff. The main findings indicated that neither emotional intelligence nor job stress consistently predicted clinical decision‐making. Although job stress demonstrated a weak positive association in the unadjusted model, this finding should be interpreted cautiously because the overall regression model was not statistically significant, and the association was not maintained after adjustment for covariates. However, a weak but statistically significant positive correlation was observed between emotional intelligence and job stress. A notable characteristic of the study population was the predominance of male participants (91.6%), reflecting the structural composition of prehospital emergency medical services in Iran. This gender imbalance has been reported in previous EMS studies in similar settings and may limit the generalizability of the findings, as gender differences in emotional regulation, coping strategies, and decision‐making styles have been documented in healthcare research [17].

The absence of statistically significant associations should be interpreted in light of several methodological considerations. The regression models explained only a small proportion of the variance in clinical decision‐making, suggesting that important determinants of clinical decision‐making were not captured in the present study. First, measurement‐related factors may have influenced the findings. The emotional intelligence instrument used in this study may not have fully captured context‐specific dimensions of emotional processing relevant to prehospital clinical decision‐making. In addition, restricted variability in the measured constructs may have attenuated the observed associations and limited the ability to detect meaningful relationships [18]. Second, the observed null findings may have been influenced by unmeasured confounding variables. Factors such as coping strategies, individual resilience, and organizational climate were not assessed but are likely to influence stress perception and decision‐making performance. These variables may also function as mediators or moderators in the relationship between emotional intelligence, job stress, and clinical decision‐making [19, 20]. The cultural and organizational context may also be relevant. Prehospital emergency personnel operate within structured hierarchical systems where organizational expectations and standardized clinical protocols may exert a stronger influence on clinical decision‐making than individual psychological traits [10]. However, this explanation remains hypothetical because these organizational factors were not directly measured in the present study.

Recent literature in emergency medical services has highlighted moral distress as an important dimension of occupational stress in prehospital care [21]. Moral distress occurs when clinicians are constrained from acting in accordance with their ethical judgments due to organizational or situational limitations. Such factors may influence clinical decision‐making beyond what is captured by general measures of job stress or emotional intelligence. Therefore, the absence of statistically significant associations in this study may partly be related to unmeasured constructs such as moral distress; however, these factors were not directly assessed [22]. The lack of significant differences across demographic variables suggests that individual characteristics alone may not fully explain variability in clinical decision‐making. However, interpretations involving broader constructs such as organizational support, leadership, workload, and team dynamics should be considered as hypotheses rather than confirmed explanations, as these factors were not directly measured in this study. In contrast to some previous studies reporting detrimental effects of job stress on clinical decision‐making, the present study did not provide robust evidence supporting such an association [23]. Although job stress demonstrated a weak positive association in the unadjusted analysis, this finding should be interpreted cautiously because the overall regression model was not statistically significant, and the association was no longer evident after adjustment for potential confounders. One possible explanation is that individuals working in high‐stress EMS environments may develop adaptive coping mechanisms over time, which could attenuate the adverse effects of stress on cognitive performance. This interpretation is consistent with resilience‐based theoretical frameworks; however, resilience was not directly measured in this study, and therefore this explanation remains speculative [24, 25].

Overall, the present findings are consistent with previous research suggesting that the relationships among job stress, emotional intelligence, and clinical decision‐making are complex and may be influenced by multiple individual, organizational, and contextual factors that were not fully captured in the present study [26, 27]. In contrast to the present findings, Ayed et al. reported a significant positive relationship between emotional intelligence and clinical decision‐making among NICU nurses using the SSEIT. Differences in clinical settings, professional roles, workload characteristics, and organizational contexts may contribute to these inconsistent findings; however, these explanations remain hypothetical because such factors were not directly measured in the present study [28]. Prior studies have also emphasized the potential role of team dynamics, leadership, and resilience in shaping clinical performance [29, 30]. However, in the present study, these factors should be interpreted as potential explanatory hypotheses for future research rather than confirmed determinants of the observed outcomes. From a theoretical perspective, the results suggest that clinical decision‐making in high‐pressure EMS environments may not be primarily determined by individual psychological traits alone, but may instead be influenced by multi‐level systems involving organizational and contextual factors. Nevertheless, due to the cross‐sectional design and lack of direct measurement of these variables, such interpretations should remain hypothesis‐driven. From a practical perspective, the limited explanatory power of the regression models suggests that emotional intelligence and job stress alone are unlikely to adequately explain variation in clinical decision‐making among EMS personnel. Consequently, future intervention strategies may benefit from considering additional individual, organizational, and contextual determinants of clinical decision‐making. However, this proposition remains hypothesis‐generating and requires confirmation in prospective and interventional studies. From an international perspective, these findings contribute to the growing body of EMS literature suggesting that psychological predictors of clinical decision‐making may vary across organizational and cultural contexts. Differences in workload patterns, training systems, healthcare structures, and organizational environments may influence the extent to which such associations are observed [31, 32]. Taken together, the present findings suggest that clinical decision‐making in prehospital emergency settings is likely influenced by multiple interacting individual, organizational, and contextual factors. Future longitudinal studies incorporating broader psychosocial and organizational variables are needed to better explain variation in clinical decision‐making and to clarify the mechanisms underlying these relationships.

## 5 Limitation

This study has several limitations that should be considered when interpreting the findings. First, the cross‐sectional design limits the ability to draw causal inferences regarding the relationships among job stress, emotional intelligence, and clinical decision‐making. Second, the study was conducted within a specific regional and organizational context, which may limit the generalizability of the findings to other settings or healthcare systems. Third, the sample was predominantly male, which may restrict the applicability of the results to more gender‐balanced populations. Fourth, data were collected using self‐reported questionnaires, which may be subject to social desirability and common‐method bias. Although anonymity was ensured, the possibility of response bias cannot be fully excluded. Finally, clinical decision‐making was assessed using a self‐reported measure rather than objective performance‐based evaluations. The absence of objective indicators may limit the accuracy of assessing actual clinical decision‐making ability. Additionally, this study did not assess other potentially relevant factors, such as organizational characteristics, resilience, coping strategies, and adherence to clinical protocols, which may influence clinical decision‐making and stress responses among EMS personnel.

## 6 Conclusion

This study found moderate levels of job stress, emotional intelligence, and clinical decision‐making among prehospital emergency medical staff. Overall, neither emotional intelligence nor job stress emerged as robust predictors of clinical decision‐making. Although job stress showed a weak positive association in the unadjusted analysis, this finding was not maintained after adjustment for potential confounders, and the overall regression models were not statistically significant. The limited explanatory power of the regression models further suggests that emotional intelligence and job stress alone are insufficient to explain variation in clinical decision‐making among EMS personnel. Future research should incorporate broader individual, organizational, and contextual factors, using longitudinal and prospective designs, to better understand the determinants of clinical decision‐making in prehospital emergency settings.

## Author Contributions

**Payam Emami:** investigation, writing – review and editing, project administration, software, supervision, writing – original draft. **Mahsa Sadafi:** conceptualization, investigation, methodology, software, data curation, project administration. **Ameneh Marzban:** software, methodology, validation, formal analysis, resources, writing – review and editing. **Nioosha Fatahi:** software, formal analysis, resources, supervision, data curation, visualization, writing – review and editing, writing – original draft, investigation, project administration.

## Funding

The authors have nothing to report.

## Conflicts of Interest

The authors declare no conflicts of interest.

## Transparency Statement

The lead author Payam Emami affirms that this manuscript is an honest, accurate, and transparent account of the study being reported; that no important aspects of the study have been omitted; and that any discrepancies from the study as planned (and, if relevant, registered) have been explained.

## Data Availability

The data supporting the findings of this study are available from the corresponding author upon reasonable request. Nioosha Fatahi had full access to all of the data in this study and takes complete responsibility for the integrity of the data and the accuracy of the data analysis.

## References

[1] P. Emami , M. Boozari Pour , H. Zahednezhad , L. Khanali Mojen , and V. Naseri , “Investigating the Relationship Between Workplace Stressors and Caring Behaviours of Nursing Staff in Inpatient Wards: A Cross‐Sectional Study,” Journal of Advanced Nursing 78, no. 4 (2022): 1066–1074. 34642973 10.1111/jan.15080

[2] A. Ghorbanian , M. Bahadori , and M. Nejati , “The Relationship Between Managers' Leadership Styles and Emergency Medical Technicians' Job Satisfaction,” Australasian Medical Journal 5, no. 1 (2012): 1. 22905048 10.4066/AMJ.2011892 PMC3413924

[3] A. Dadashzadeh , A. Rahmani , H. Hasankhani , and S. Elmi , “Work‐Related Burden of Stress Among Emergency Medical Service Personnel,” Journal of Practical Emergency Medicine 5, no. 1 (2018): e2, 10.22037/ijem.v2i1.18550.

[4] S. Valiei , M. Rezaei , and K. Rezaei , “The Relationship Between Personality Characteristics and Nursing Occupational Stress,” Iranian Journal of Psychiatric Nursing 1, no. 3 (2013): 27–34.

[5] A. Dadmehrnia , S. A. A. Faghihi , H. R. Koohestani , G. Pourbairamian , and M. H. Keshavarzi , “An Elucidation on the Challenges of Pre‐Hospital Emergency Technicians: A Study on Emergency Medical Technicians in Shiraz, Iran,” Shiraz E‐Medical Journal 24, no. 8 (2023): e137606, 10.5812/semj-137606.

[6] G. Babamiri and Z. Karimiankakolaki , “Investigating the Impact of Crisis Management Training on Clinical Decision‐Making and Management of Stress Factors in the Personnel of Emergency Medical Services: Randomized Controlled Trial,” BMC Medical Education 25, no. 1 (2025): 1180. 40836232 10.1186/s12909-025-07730-6 PMC12366106

[7] R. Cheraghi , N. Parizad , V. Alinejad , M. Piran , and L. Almasi , “The Effect of Emotional Intelligence on Nurses' Job Performance: the Mediating Role of Moral Intelligence and Occupational Stress,” BMC Nursing 24, no. 1 (2025): 130. 39905367 10.1186/s12912-025-02744-3 PMC11796207

[8] M. Almeida , C. Lobão , A. Coelho , and V. Parola , “Emotional Management Strategies in Prehospital Nurses: A Scoping Review,” Nursing Reports 13, no. 4 (2023): 1524–1538. 37987407 10.3390/nursrep13040128 PMC10661275

[9] U. Andersson , H. Maurin Söderholm , B. Wireklint Sundström , M. Andersson Hagiwara , and H. Andersson , “Clinical Reasoning in the Emergency Medical Services: An Integrative Review,” Scandinavian Journal of Trauma, Resuscitation and Emergency Medicine 27, no. 1 (2019): 76. 31426839 10.1186/s13049-019-0646-y PMC6700770

[10] E. Bai , Z. Zhang , Y. Xu , X. Luo , and K. Adelgais , “Enhancing Prehospital Decision‐Making: Exploring User Needs and Design Considerations for Clinical Decision Support Systems,” BMC Medical Informatics and Decision Making 25, no. 1 (2025): 31. 39825293 10.1186/s12911-024-02844-1 PMC11742207

[11] J. B. King , Y. Li , N. A. Gillespie , and N. M. Ashkanasy , “Emotional Intelligence Training Improves Stress Regulation and Performance in High‐Stress Occupations,” Scientific Reports 16 (2026): 6673. 41618102 10.1038/s41598-026-36216-8 PMC12914035

[12] S. Hashmi , O. Tahir , Z. Nasir , et al., “Impact of Emotional Intelligence on Professional Performance and Stress Resilience Among Healthcare Practitioners,” Cureus 16, no. 11 (2024): e74113, 10.7759/cureus.74113. 39712679 PMC11662091

[13] A. Khazaei , A. Afshari , R. Salimi , A. Fattahi , B. Imani , and M. Torabi , “Exploring Stress Management Strategies Among Emergency Medical Service Providers in Iran: A Qualitative Content Analysis,” BMC Emergency Medicine 24, no. 1 (2024): 106. 38926678 10.1186/s12873-024-01024-8 PMC11209986

[14] M. E. Musio , F. Ginogi , S. Casini , et al., “The Impact of Emotional Intelligence on Nurses' Professional Quality of Life in Pre‐Hospital Emergency Settings: A Multicentre Mixed‐Method Study,” Journal of Clinical Nursing 34, no. 1 (2025): 108–116. 39428361 10.1111/jocn.17511 PMC11655433

[15] R. Cheraghi , L. Almasi , V. Alinejad , N. Aghakhani , M. Jasemi , and S. Eghtedar , “The Relationship Between Emotional Intelligence With Job Performance and Occupational Stress in Nurses Working in Educational and Medical Centers in Urmia in 2022,” Nursing And Midwifery Journal 21, no. 7 (2023): 575–588.

[16] N. S. Schutte , J. M. Malouff , L. E. Hall , et al., “Development and Validation of a Measure of Emotional Intelligence,” Personality and Individual Differences 25, no. 2 (1998): 167–177.

[17] M. A. Marzaleh , M. Peyravi , E. Ahmadi , H. Mahmoodi , I. Shakibkhah , and H. Armin , “Lived Experiences of Iranian Prehospital Emergency Technicians in Facing Women's Emergencies: A Phenomenological Study,” BMC Emergency Medicine 24, no. 1 (2024): 98. 38858640 10.1186/s12873-024-01019-5 PMC11165769

[18] L. Tang , W. Yue , and X. Ma , “Emotional Intelligence and Clinical Decision‐Making Confidence in Nurses: The Chain Mediating Effect of Creative Self‐Efficacy and Self‐Directed Learning,” Frontiers in Medicine 12 (2025): 1708634. 41451101 10.3389/fmed.2025.1708634 PMC12730166

[19] B. Kunas , H. Gruber , and A.‐R. Laireiter , “Moderated Mediations of Occupational Stress on Mental Health: The Different Roles of Resilience and Coping,” Current Psychology 45, no. 3 (2026): 302.

[20] P. Pratama and M. Sampe Tondok , “Resilience as a Moderator of Perceived Stress and Coping Strategies in Sophomore College Students post‐COVID‐19 Pandemic,” Bulletin of Counseling and Psychotherapy 5, no. 2 (2023): 251–260.

[21] D. Sedláček , A. Lochmannová , R. Šín , J. Křivková , K. Kovalčinová , and P. Marek , “Frontline Ethical Burdens: A Mixed‑Methods Investigation of Moral Distress in Emergency Medical Services,” Bratislava Medical Journal 126, no. 7 (2025): 1463–1471.

[22] M. Buchbinder , A. Browne , N. Berlinger , T. Jenkins , and L. Buchbinder , “Moral Stress and Moral Distress: Confronting Challenges in Healthcare Systems under Pressure,” American Journal of Bioethics 24, no. 12 (2024): 8–22. 10.1080/15265161.2023.2224270 PMC10758677 37347222

[23] A. J. Porcelli and M. R. Delgado , “Stress and Decision Making: Effects on Valuation, Learning, and Risk‐Taking,” Current Opinion in Behavioral Sciences 14 (2017): 33–39. 28044144 10.1016/j.cobeha.2016.11.015 PMC5201132

[24] E. Jacobs and R. J. Keegan , “Sustaining Optimal Performance When the Stakes Could Not Be Higher: Emotional Awareness and Resilience in Emergency Service Personnel (With Learnings for Elite Sport),” Frontiers in Psychology 13 (2022): 891585. 36118503 10.3389/fpsyg.2022.891585 PMC9472212

[25] M. M. Alqahtani , N. M. Alotaibi , A. A. Alsahabi , et al., “Psychological Resilience and Coping Strategies Among Emergency Medical Technicians: A Systematic Review 2015–2025,” Review of Diabetic Studies 2025 (2025): 547–559.

[26] N. Jawabreh , “The Relationship Between the Emotional Intelligence and Clinical Decision Making Among Nursing Students,” SAGE Open Nursing 10 (2024): 23779608241272459. 39119200 10.1177/23779608241272459 PMC11307361

[27] C. K. Fallon , G. Matthews , A. R. Panganiban , R. Wohleber , and R. D. Roberts , “Emotional Intellegence and Decision Making under Stress,” Proceedings of the Human Factors and Ergonomics Society Annual Meeting 57, no. 1 (2013): 873–877.

[28] A. Ayed , I. Aqtam , M. Z. Malak , et al., “Insights into the Relationship Between Emotional Intelligence and Critical Thinking Among Nursing Students,” BMC Nursing 24, no. 1 (2025): 1107. 40849675 10.1186/s12912-025-03782-7 PMC12374380

[29] G. Fernández Castillo , M. Khan , L. Berger , R. Linhardt , T. Jean‐Baptiste , and E. Salas , “The Team Resilience Prescription: Navigating Adaptive and Maladaptive Processes in Healthcare Teams,” Journal of Interprofessional Care 39, no. 2 (2025): 314–320. 39955780 10.1080/13561820.2025.2460477 PMC11932645

[30] H. Yang and S. Suntrayuth , “Enhancing Team Resilience Through Team Knowledge Dynamics: A Mediated Approach,” Frontiers in Psychology 16 (2025): 1615909. 40978252 10.3389/fpsyg.2025.1615909 PMC12443702

[31] H. Skundberg‐Kletthagen and K. Kirbakk Fjær , “Exploring Competence in Mental Health Crisis Management: A Qualitative Study of Norwegian Prehospital Emergency Medical Personnel,” BMJ Open 15, no. 10 (2025): e098877. 10.1136/bmjopen-2025-098877 PMC12506131 41057186

[32] S. Lawn , L. Roberts , E. Willis , L. Couzner , L. Mohammadi , and E. Goble , “The Effects of Emergency Medical Service Work on the Psychological, Physical, and Social Well‐Being of Ambulance Personnel: A Systematic Review of Qualitative Research,” BMC Psychiatry 20, no. 1 (2020): 348. 32620092 10.1186/s12888-020-02752-4 PMC7332532

